# Different effects of smoking on atopic and non‐atopic adult‐onset asthma

**DOI:** 10.1002/clt2.12072

**Published:** 2021-10-11

**Authors:** Taina K. Lajunen, Jouni J. K. Jaakkola, Maritta S. Jaakkola

**Affiliations:** ^1^ Center for Environmental and Respiratory Health Research University of Oulu Oulu Finland; ^2^ Biocenter University of Oulu Oulu Finland; ^3^ Medical Research Center University of Oulu Oulu Finland; ^4^ Finnish Meteorological Institute Helsinki Finland

**Keywords:** asthma, epidemiology, prevention, smoking, tobacco

## Abstract

**Background:**

Both tobacco smoking and atopy increase the risk of adult‐onset asthma. We studied if there are differences in the effects of smoking on the risks of atopic and non‐atopic adult‐onset asthma, and if gender modifies these effects.

**Methods:**

The Finnish Environment and Asthma Study (FEAS) includes 521 incident cases of adult‐onset asthma and 932 population‐based controls, aged 21 to 63 years, recruited from a geographically defined area of Pirkanmaa, South Finland. Asthma was defined based on symptoms and lung function measurements, atopy by IgE antibodies to common aeroallergens and smoking by the study questionnaire.

**Results:**

Altogether 212 cases were atopic, and 251 cases were non‐atopic. Regular smoking increased the risk of atopic asthma (adjusted OR 1.24, 95% CI 0.83–1.85), this effect was seen in women (aOR 1.77, 1.06–2.95) but not in men (aOR 0.75, 0.39–1.45). Among regular smokers, the amount smoked was lowest among women with atopic asthma. Recent quitting of smoking was related to increased risk of both atopic (aOR 4.91, 2.26–10.65) and non‐atopic (aOR 4.37, 1.87–10.21) asthma. Having quitted smoking over a year ago was related to increased risk of non‐atopic asthma (aOR 1.57, 1.08–2.28), mainly in men (aOR 2.03, 1.06–3.88).

**Conclusions:**

In women, rather small amounts of regular smoking increase the risk of atopic asthma. However, for non‐atopic asthma, the smoking induced risk continues for longer after quitting, especially in men. In conclusion, the effects of smoking on the risks of atopic and non‐atopic asthma differ, and gender modifies these effects.

## 1 INTRODUCTION

Tobacco smoking is common worldwide. The age standardised WHO estimate of the prevalence of current smoking in 2018 was 18.9% globally and 26.2% in Europe.^1^ Tobacco smoke may affect both type 1 and type 2 immune responses, in both stimulatory and inhibitory manner.^2^, ^3^, ^4^ In addition, factors such as gender, age and viral infections modify these effects.^2^, ^3^, ^4^ Tobacco smoke has been shown to increase mucus production and permeability of respiratory epithelium, impair mucociliary clearance, upregulate oxidative responses, and be associated with increased airway hyperresponsiveness (AHR), severe asthma symptoms and decline in lung function.^2^, ^4^

We have previously reported from the Finnish Environment and Asthma Study (FEAS) that both current smoking (OR 1.33, 95% CI 1.00–1.77) and previous smoking (OR 1.49, 1.12–1.97) increase the risk of adult‐onset asthma.^5^ Several other studies have confirmed these findings.^6^, ^7^, ^8^, ^9^, ^10^, ^11^, ^12^, ^13^, ^14^, ^15^, ^16^, ^17^, ^18^, ^19^, ^20^, ^21^, ^22^, ^23^, ^24^, ^25^, ^26^, ^27^ Moreover, we observed in the FEAS that the risk of adult‐onset asthma related to current or past smoking was about 2.4 times higher in women compared to never smoking men.^5^ Thus, our previous results suggested that women are more susceptible to the harmful effects of smoking. Findings compatible with ours have been reported by others.^9^, ^14^, ^16^, ^19^, ^22^ In addition, we have reported from the FEAS^28^ along with other studies^6^, ^9^, ^10^, ^14^, ^17^, ^18^, ^21^, ^24^, ^27^, ^29^, ^30^, ^31^, ^32^ that atopy and allergic diseases are risk factors for adult‐onset asthma. However, there are only a few previous studies that have investigated potentially different effects of smoking on the risk of atopic and non‐atopic adult‐onset asthma.^10^, ^14^, ^17^, ^32^ The results of these studies are somewhat inconsistent, and they did not address potential modifying effect by gender. Our objective was to elaborate potentially different effects of personal smoking on the risk of atopic and non‐atopic adult‐onset asthma subtypes, and to evaluate if such relations are modified by gender. Further information on the role of atopy and gender in smoking and asthma would support personalised approach to prevent taking up smoking and to motivate quitting.

## 2 METHODS

### 2.1 Study subjects

The detailed description of the recruitment of cases and controls has been published elsewhere.^5^, ^28^ We systematically recruited all new cases of adult‐onset asthma 21–63 years old in 1997–2000 in the Pirkanmaa Hospital District, South Finland. We selected as cases only those with no previously diagnosed asthma or previous long‐term use of any asthma medication. A total of 521 cases participated (response rate 86%). The control subjects were randomly drawn from the source population. A total of 1016 control subjects participated (response rate 80%). After excluding those with previous or current asthma, those older than 63 years, and those returning incomplete questionnaire, our study population included 932 controls. The study was approved by the ethics committees of the Finnish Institute of Occupational Health and the Tampere University Hospital.

### 2.2 Study design

This was a population‐based incident case‐control study of adult‐onset asthma.^5^, ^28^ The source population consisted of adults 21–63 years of age living in a geographically defined area of Pirkanmaa in South Finland. The population of the study area was 447,051 in 2000.

#### 2.2.1 Questionnaire

When entering the study, the subjects answered a self‐administered questionnaire enquiring about their personal characteristics, health information, active smoking, second‐hand smoke exposure, occupation, work environment, home environment, and diet.^5^, ^28^ Smoking was categorised in the following way: regular, ≥1 cigarette or cigar a day or ≥25 g of pipe tobacco a month; occasional, <1 cigarette a day or <25 g of pipe tobacco a month; current: regular and occasional smokers; previous: quit smoking either <12 months ago (=recent quitting) or >1 year ago. The cumulative life‐time consumption of tobacco was estimated as cigarette‐years, calculated as the average smoking rate (cigarettes/cigars/pipefuls per day) × duration of smoking.

#### 2.2.2 Diagnosis of asthma, lung function measurements, and definition of atopy

The details of diagnosis of asthma and lung function measurements^5^, ^28^ as well as definition of atopy^28^ have been published previously. Shortly, the diagnostic criteria for asthma were (1) occurrence of at least one asthma‐related symptom, and (2) demonstration of airways obstruction with significant reversibility in lung function investigations. Spirometry and bronchodilatation test were recorded with a pneumotachograph spirometer using disposable flow transducer (Medikro 905, Medikro) according to the standards of the American Thoracic Society.^33^ Presence of obstruction was defined based on the reference values derived from the Finnish population.^34^ For this study, we excluded 26 individuals who were found to have asthma‐COPD overlap syndrome (ACOS).^35^ Atopy was defined based on presence of specific IgE antibodies to common aeroallergens, including birch, timothy grass, mugwort, cat, dog, horse, *Dermatophagoides pteronyssinus*, and/or *Aspergillus fumigatus* in serum.^28^ The results were expressed as Phadiatop scores from 0 to 6. A positive score (≥1) corresponded to the limit of ≥0.35 kU/L and was used to define atopy. The Phadiatop measurements were available for 463 cases, which is the final number of cases included in this substudy. There were no significant differences in the distribution of the main characteristics between the 463 cases included in this substudy and the 32 cases who did not give a blood sample and thus have no Phadiatop measurement (Table S1).

#### 2.2.3 Statistical analysis

We analysed potential effect of current and former smoking on the risk of atopic and non‐atopic adult‐onset asthma, with never smokers forming the reference category. Next, we considered the regularity of current smoking (occasional or regular) and among ex‐smokers, the time since quitting smoking (i.e. ≤12 months ago or >1 year ago). We adjusted the analyses for gender, age, education (as an indicator of socioeconomic status), pets indoors, work exposures except for moulds, and mould exposure at work or at home as potential confounders. In addition, we studied if gender modifies these relations by performing gender‐stratified analyses. We applied multinomial logistic regression analysis for all models. We also compared the years smoked, daily smoking rate, duration since smoking cessation, and cigarette‐years across the categories of asthma subtypes and by gender applying Kruskal–Wallis test with Dwass, Steel, Critchlow–Fligner multiple comparisons post‐hoc procedure. We used SAS 9.4 for all statistical analyses (SAS Institute Inc.).

## 3 RESULTS

### 3.1 Characteristics

Those with atopic asthma reported more often pets indoors now or previously than those with non‐atopic asthma or the controls (Table 1). In contrast, those with non‐atopic asthma were more often women, were older and had a lower level of education than those with atopic asthma or the controls.

**TABLE 1:** Characteristics of the study population

Characteristic	Controls, *n* (%)	Non‐atopic asthma, *n* (%)	Atopic asthma, *n* (%)
Total, *n*	932	251	212
Gender			
Men	438 (47.0)	69 (27.5)	75 (35.4)
Women	494 (53.0)	182 (72.5)	137 (64.6)
Age, years			
21–29	141 (15.1)	22 (8.8)	78 (36.8)
30–39	224 (24.0)	42 (16.7)	55 (25.9)
40–49	254 (27.3)	72 (28.7)	42 (19.8)
50–59	240 (25.8)	90 (35.9)	29 (13.7)
60–63	73 (7.8)	25 (10.0)	8 (3.81)
Education^a^			
No vocational schooling	154 (16.6)	56 (22.5)	32 (15.1)
Vocational course	104 (11.2)	54 (21.7)	22 (10.4)
Vocational institution	271 (29.2)	63 (25.3)	75 (35.4)
College‐level education	261 (28.1)	54 (21.7)	51 (24.1)
University or corresponding	138 (14.9)	22 (8.8)	32 (15.1)
Pets at home sometimes	616 (66.1)	164 (65.3)	164 (77.4)
Any work exposure^b^	579 (62.1)	155 (61.8)	129 (60.9)
Indoor mould exposure at work or at home^c^	193 (20.7)	55 (21.9)	51 (24.1)
Smoking^d^			
Never	485 (52.2)	118 (47.6)	104 (49.1)
Previous^e^	205 (22.0)	71 (28.6)	47 (22.2)
Quit >1 year ago	188 (20.3)	60 (24.2)	30 (14.2)
Quit <12 months ago	15 (1.6)	11 (4.4)	17 (8.0)
Current	240 (25.8)	59 (23.8)	61 (28.8)
Occasional	50 (5.4)	19 (7.7)	8 (3.8)
Regular	190 (20.4)	40 (16.1)	53 (25.0)

Abbreviation: NA, Not applicable.

^a^ Education missing for four controls and two cases with non‐atopic asthma.

^b^ Self‐reported exposure to sensitisers, dusts and/or fumes.

^c^ Mould exposure missing (answered ‘do not know’) for one control.

^d^ Smoking missing for two controls and three cases with non‐atopic asthma.

^e^ Time of quitting smoking missing for two controls.

### 3.2 The effects of smoking on the risk of asthma subtypes

#### 3.2.1 Current smoking

**FIGURE 1:**
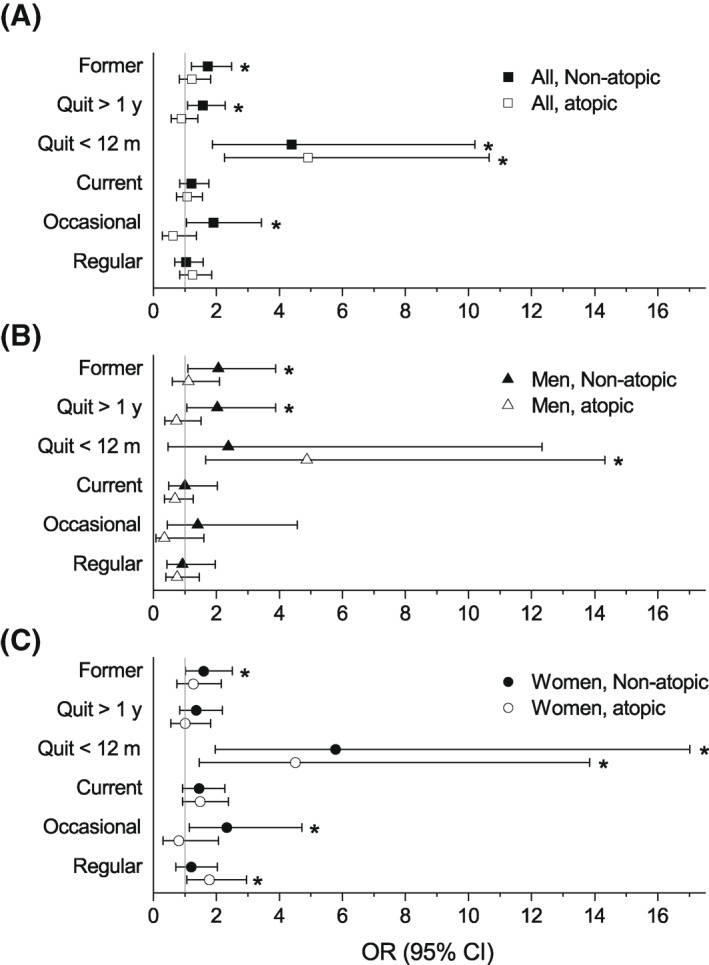
The effect of smoking status on non‐atopic (black fill) and atopic (no fill) adult‐onset asthma, in the whole study population (A) (squares), and in men (B) (triangles) and in women (C) (circles) separately, when compared to the never‐smokers. The statistically significant effects are indicated by asterisks. The multinomial regression analyses were adjusted for gender (the whole study population only), age, education, pets at home, work exposures, and indoor moulds

Current smoking was found to have little effect on the risk of either asthma subtype in the whole study population, and in men (Figure 1A,B, Tables 2 and 3). In women, the risk was somewhat increased for both atopic (OR 1.48, 0.92–2.38) and non‐atopic (OR 1.44, 95% CI 0.92–2.27) asthma, but these were not statistically significant (NS) (Figure 1C, Table 3).

**TABLE 2:** The effect of smoking status on non‐atopic and atopic asthma, among adults with new‐onset asthma

Smoking status	Controls	Non‐atopic asthma					Atopic asthma				
	*N* = 932^a^	*N* = 251^a^					*N* = 212				
			Unadjusted		Adjusted^b^ ^,^ ^c^			Unadjusted		Adjusted^b^ ^,^ ^c^	
	*n*	*n*	OR	95% CI	OR	95% CI	*n*	OR	95% CI	OR	95% CI
Never smoker	485	118	Ref	‐	Ref	‐	104	Ref	‐	Ref	‐
Former smoker	205	71	**1.42**	**1.02**–**1.99**	**1.73**	**1.21**–**2.48**	47	1.07	0.73–1.57	1.22	0.82–1.82
Quit >1 year ago	188^d^	60	1.31	0.91–1.87	**1.57**	**1.08**–**2.28**	30	0.74	0.48–1.16	0.89	0.56–1.41
Quit <12 months ago	15^d^	11	**3.01**	**1.35**–**6.73**	**4.37**	**1.87**–**10.21**	17	**5.29**	**2.56**–**10.92**	**4.91**	**2.26**–**10.65**
Current smoker	240	59	1.01	0.71–1.43	1.21	0.84–1.76	61	1.19	0.83–1.69	1.07	0.73–1.56
Occasional smoker	50	19	1.56	0.89–2.75	**1.90**	**1.05**–**3.43**	8	0.59	0.27–1.32	0.62	0.28–1.37
Regular smoker	190	40	0.87	0.58–1.29	1.04	0.68–1.58	53	1.30	0.90–1.89	1.24	0.83–1.85

*Note*: The statistically significant effects are shown bolded.

Abbreviations: CI, Confidence interval; OR, odds ratio; Ref, reference category.

^a^ Smoking missing for two controls and three cases with non‐atopic asthma.

^b^ Adjusted for gender, age, education, pets at home, work exposures, and indoor moulds.

^c^ Indoor mould exposure missing (answered ‘do not know’) for one control.

^d^ Time of quitting smoking missing for two previous smoking controls.

**TABLE 3:** The effect of smoking status on non‐atopic and atopic asthma in men and in women, among adults with new‐onset asthma

Smoking status	Controls	Non‐atopic asthma					Atopic asthma				
	*N* = 932^a^	*N* = 251^a^					*N* = 212				
			Unadjusted		Adjusted^b^ ^,^ ^c^			Unadjusted		Adjusted^b^ ^,^ ^c^	
	*n*	*n*	OR	95% CI	OR	95% CI	*n*	OR	95% CI	OR	95% CI
Men, total	438^a^	69^a^					75				
Never smoker	171	19	Ref	‐	Ref	‐	32	Ref	‐	Ref	‐
Former smoker	122	31	**2.29**	**1.24**–**4.24**	**2.06**	**1.09**–**3.88**	22	0.96	0.53–1.74	1.12	0.60–2.10
Quit >1 year ago	114	29	**2.29**	**1.23**–**4.28**	**2.03**	**1.06**–**3.88**	13	0.61	0.31–1.21	0.73	0.36–1.51
Quit <12 months ago	8	2	2.25	0.45–11.37	2.38	0.46–12.34	9	**6.01**	**2.16**–**16.75**	**4.87**	**1.66**–**14.32**
Current smoker	144	18	1.13	0.57–2.24	1.00	0.49–2.03	21	0.78	0.43–1.41	0.69	0.35–1.26
Occasional smoker	25	4	1.44	0.45–4.57	1.41	0.44–4.57	2	0.43	0.10–1.90	0.35	0.08–1.60
Regular smoker	119	14	1.06	0.51–2.20	0.92	0.43–1.96	19	0.85	0.46–1.58	0.75	0.39–1.45
Women, total	494^a^	182^a^					137				
Never smoker	314	99	Ref	‐	Ref	‐	72	Ref	‐	Ref	‐
Former smoker	83	40	1.53	0.99–2.37	**1.59**	**1.02**–**2.50**	25	1.31	0.79–2.20	1.26	0.74–2.15
Quit >1 year ago	74^d^	31	1.33	0.83–2.14	1.35	0.83–2.19	17	1.00	0.56–1.80	1.00	0.55–1.81
Quit <12 months ago	7^d^	9	**4.08**	**1.48**–**11.23**	**5.77**	**1.96**–**17.02**	8	**4.98**	**1.75**–**14.19**	**4.50**	**1.46**–**13.84**
Current smoker	96	41	1.36	0.88–2.08	1.44	0.92–2.27	40	**1.82**	**1.16**–**2.85**	1.48	0.92–2.38
Occasional smoker	25	15	1.90	0.97–3.75	**2.32**	**1.14**–**4.71**	6	1.05	0.41–2.65	0.80	0.30–2.06
Regular smoker	71	26	1.16	0.70–1.92	1.20	0.71–2.03	34	**2.09**	**1.29**–**3.38**	**1.77**	**1.06**–**2.95**

*Not*e: The statistically significant effects are shown bolded.

Abbreviations: CI, confidence interval; OR, odds ratio; Ref, reference category.

^a^ Smoking missing for one control and one case with non‐atopic asthma in men, and for one control and two cases with non‐atopic asthma in women.

^b^ Adjusted for age, education, pets at home, work exposures and indoor moulds.

^c^ Mould exposure missing (answered ‘do not know’) for one control in women.

^d^ Time of quitting smoking missing for two previous smoking control women.

When we considered the regularity of smoking, occasional smoking had no effect on atopic asthma but increased the risk of non‐atopic asthma in the whole study population (OR 1.90, 1.05–3.43) and especially in women (OR 2.32, 1.14–4.71) (Figure 1A–C, Tables 2 and 3). Regular smoking, on the other hand, increased the risk of atopic asthma in women (OR 1.77, 1.06–2.95). For non‐atopic asthma in women, the effect estimate of regular smoking was somewhat elevated, but NS (OR 1.20, 0.71–2.03).

#### 3.2.2 Former smoking

In comparison to never smoking former smoking associated with somewhat elevated (OR 1.22, 0.82–1.82), but NS, risk of atopic asthma. Whereas the risk of non‐atopic asthma was increased (OR 1.73, 95% CI 1.21–2.48) (Figure 1A, Table 2). Analysing the genders separately did not reveal many differences (Figure 1B,C, Table 3).

When considering the time since quitting smoking, we found recent quitting (i.e. less than 12 months ago) to be related to considerably increased risk of both asthma subtypes, with an OR 4.91 (2.26–10.65) for atopic asthma and an OR 4.37 (95% CI 1.87–10.21) for non‐atopic asthma (Figure 1A, Table 2). Stratification by gender did not influence the results much, except that the risk of non‐atopic asthma in men was NS (Figure 1B,C, Table 3).

Having quitted smoking over a year ago did not affect the risk of atopic asthma neither in the whole study population (OR 0.89, 0.56‐1.41), nor in the analyses by gender (Figure 1A–C, Tables 2 and 3). In contrast, the risk of non‐atopic asthma was increased in the whole study population (OR 1.57, 1.08–2.28), and in men (OR 2.03, 1.06–3.88) (Figure 1A–C, Tables 2 and 3).

#### 3.2.3 Smoking history

We next analysed if there are differences in smoking habits that could explain the differences observed in the risk of the asthma subtypes (Table 4). Among those, who had quitted smoking over a year ago, men with non‐atopic asthma had generally smoked more, that is, had higher median smoking rate and cigarette‐years, than women with non‐atopic or atopic asthma. Among recent quitters and occasional smokers, there were no statistically significant differences in smoking variables according to asthma subtype or gender. Among regular smokers, men with non‐atopic asthma had a longer history of smoking (in years smoked) and had smoked more (in terms of both smoking rate and cigarette‐years) than women with atopic asthma. In addition, women with non‐atopic asthma had smoked more (in cigarette‐years) than women with atopic asthma.

**TABLE 4:** Comparison of smoking related variables between non‐atopic and atopic asthma by smoking status

Smoking status	Variable	Men						Women						*p*‐value^c^	Pairwise multiplecomparison of the categories^d^
		Non‐atopic asthma			Atopic asthma			Non‐atopic asthma			Atopic asthma				
		*N* = 69^a^			*N* = 75			*N* = 182^b^			*N* = 137				
		*N*	Md	Q1–Q3	*N*	Md	Q1–Q3	*N*	Md	Q1–Q3	*N*	Md	Q1–Q3		
Quit >1 year ago	Years since quitting	29	9.0	4.0–22.5	13	6.0*^1^	4.0–11.0	31	18.0*^1^	10.0–20.0	17	8.0	3.0–19.0	0.07	*^1^ ***p* = 0.033**
	Years smoked		10.0	6.0–20.0		10.0	3.5–27.5		6.5	4.0–14.0		9.0	5.0–13.0	0.19	All NS
	Smoking rate cig/day		15.0*^1,^*^2^	**10.0–20.0**		**15.0**	**10.0**–**20.0**		10.0*^1^	**5.0**–**18.0**		8.0*^2^	**5.0**–**13.0**	**0.001**	*^1^ ***p* = 0.005**
															*^2^ ***p* = 0.003**
	Cigarette years		200.0*^1,^*^2^	**70.0–375.0**		**147.5**	**23.6**–**512.5**		60.0*^1^	**20.0**–**200.0**		51.8*^2^	**16.0**–**120.0**	**0.002**	*^1^ ***p* = 0.006**
															*^2^ ***p* = 0.005**
Quit <12 months ago	Months since quitting	2^e^	8.0^e^	NA	9	4.0	2.0–8.0	9	4.0	3.0–9.0	8	3.5	1.0–8.5	0.84	All NS
	Years smoked		25.0	NA		10.0	8.0–20.0		20.0	10.0–30.0		10.0	6.5–10.0	0.21	All NS
	Smoking rate cig/day		11.4	2.9–20.0		20.0	12.0–20.0		10.0	7.0–16.0		6.0	5.0–10.0	0.27	All NS
	Cigarette years		500.0	NA		180.0	160.0–216.0		200.0	70.0–300.0		80.0	35.0–115.0	0.09	All NS
Occasional smoking	Years smoked	4	19.0*^1^	16.5–22.5	2	18.5	7.0–30.0	15	10.0	4.0–15.0	6	8.0*^1^	7.0–10.0	0.14	*^1^ *p* = 0.066
	Smoking rate cig/day		2.1	0.8–4.4		1.1	0.7–1.4		1.4	0.4–2.6		1.1	0.7–2.1	0.93	All NS
	Cigarette years		39.3	19.1–81.4		23.9	5.0–42.9		6.1	2.9–22.9		11.4	4.3–11.4	0.57	All NS
Regular smoking	Years smoked	14	30.5*^1^	**20.0–40.0**	19	**20.0**	**15.0**–**30.0**	26	25.0*^2^	**19.0**–**30.0**	34	20.0*^1,^*^2^	**10.0**–**25.0**	**0.006**	*^1^ ***p* = 0.012**
															*^2^ *p* = 0.071
	Smoking rate cig/day		20.0*^1^	**13.0–25.0**		15.0*^2^	**10.0**–**20.0**		**13.5**	**8.0**–**19.0**		10.0*^1,^*^2^	**8.0**–**13.0**	**0.002**	*^1^ ***p* = 0.005**
															*^2^ *p* = 0.066
	Cigarette years		660.0*^1^	**180.0‐800.0**		**255.0**	**180.0**–**500.0**		300.0*^2^	**150.0**–**520.0**		150.0*^1,^*^2^	**70.0**–**300.0**	**0.001**	*^1^ ***p* = 0.005**
															*^2^ ***p* = 0.049**

*Note*: Statistically significant differences are shown bolded.

Abbreviations: Cig, cigarette; Md, Median; NA, Not Applicable; NS, Not Statistically Significant; Q1, lower quartile; Q3, Upper quartile.

^a^ Time since quitting missing for one man with non‐atopic asthma.

^b^ Time since quitting missing for two women with non‐atopic asthma.

^c^ Kruskal‐Wallis test for difference over gender and the asthma subtypes.

^d^ Dwass, Steel, Critchlow‐Fligner multiple comparisons post‐hoc procedure. Each two categories are compared, and the respective *p*‐values are noted with an asterisk and a number, for example, *^1^ or *^2^.

^e^ Months since quitting, years smoked and cigarette years missing for one person in this category.

## 4 DISCUSSION

In this population‐based study we show evidence that smoking affects differently the risk of developing new atopic asthma and new non‐atopic asthma in adulthood. Effects of both former and current smoking show similar pattern. We show for the first time that gender modifies these effects. This raises new questions about the underlying mechanisms of adult‐onset asthma and supports personalised approach in preventing smoking related damages.

### 4.1 Current smoking

In our pervious study, the risk of adult‐onset asthma due to occasional smoking was elevated (OR 1.25, 95% CI 0.76–2.06), but NS.^5^ Our present, more detailed analysis revealed that occasional smoking associated with an increased risk of non‐atopic asthma, but not with atopic asthma. This effect was stronger in women. On the other hand, regular smoking associated with an increase in the risk of atopic asthma, and only in women. However, in women there was also some tendency for increased risk of non‐atopic asthma. It is worth noticing that the regular smoking atopic asthmatics, and especially women, had smoked relatively small quantities when compared to the non‐atopic regular smokers. Thus, our findings imply that women may develop atopic asthma even from relatively small quantities of regular smoking. In line with our finding, an interaction between pets and smoking on asthma was reported for Canadian women, but not for men.^16^ On the other hand, in relation to occasional smoking the risk of non‐atopic asthma increased, while no such increase was detected for atopic asthma. This could be due to an individual level interplay between the strength of nicotine dependency and the severity of smoking caused symptoms. Perhaps among the most sensitive individuals, only those with strong addiction keep on smoking, while others prefer to quit smoking. In contrast, those who have less severe symptoms may prefer occasional smoking over quitting. However, the number of occasional smokers in our study was small, and thus, the interpretation of those results needs to be done with some caution. In men, current smoking had no effect on the risk of either asthma subtype. This may be explained by the ‘healthy smoker’‐concept,^36^ according to which only those, who do not develop smoking related symptoms remain smokers. Further, in women ovarian hormone fluctuations may make total quitting of smoking more difficult.^37^

### 4.2 Previous smoking

The risk of new asthma in our study was the highest among those, who had quitted smoking recently, that is, during the previous 12 months. This effect was not markedly different between atopic and non‐atopic asthma or dependent on gender. In line with this, we have previously demonstrated that recent quitting of smoking associates with decreased lung function among those with new asthma in the FEAS.^38^ The most likely explanation for the increased risk of asthma in this smoking category is that people who start to experience asthma symptoms tend to quit smoking, but this may happen too late in relation to development of asthma. In line with our findings, other studies have also reported higher risk of asthma among recent quitters, when compared to never smokers.^7^, ^12^ On the other hand, Godtfredsen et al. suggested that their study subjects could have misinterpreted their symptoms of COPD as asthma. In our study such misclassification is not likely, as we applied an objective definition of asthma based on showing obstruction in lung function measurements with significant bronchodilation response and we excluded ACOS cases from these analyses. Our findings highlight the importance of early smoking prevention and providing support for early cessation among those who have already started this habit.

After longer smoking cessation periods, our results show that the beneficial effect of quitting smoking begins to emerge, especially in relation to atopic asthma. However, among non‐atopic asthmatics we observed that the risk remained somewhat elevated and was more pronounced in men. This somewhat unexpected finding of higher risk in men could be explained by higher amount of tobacco smoked among the non‐atopic men.

### 4.3 Possible mechanisms

Among those who develop non‐atopic asthma, smoking may direct the immune responses towards T2‐low type asthma that is considered to be more innate immunity mediated, is characterised by normal levels of eosinophils, low fractional exhaled nitric oxide (FeNO), and can be neutrophilic or pauci‐granulocytic,^3^, ^4^ or even towards to early stages of ACOS. In this study, we excluded those with ACOS present already at the time of the diagnosis of asthma, but it is possible that some of the participants will develop ACOS later in life. Those with no atopic tendency may have less sensitive airways than those who develop atopic asthma, with respect to sensing the harmful effects of tobacco smoke at an early stage. Thus, they may tolerate larger amounts of smoking, as their symptoms may take longer to manifest, and onset of asthma in them may take place later despite quitting smoking. On the other hand, those who develop atopic asthma may more likely have T2‐high type asthma that is characterised by IgE‐mediated sensitisation, eosinophilia and elevated FeNO.^3^, ^4^ The airways of these individuals may thus be in a more sensitive and vulnerable state, for example, due to pre‐existing inflammation and AHR induced by allergens. Thus, their tobacco smoke induced symptoms may manifest in an earlier phase.

#### 4.3.1 Validity issues

Validity of the FEAS regarding smoking has been discussed in detail elsewhere.^5^ Shortly, in the FEAS we aimed to recruit all the new asthma cases occurring in the working‐age source population during the study period. Asthma diagnosis was based on reported respiratory symptoms and on objective lung function measurements showing obstruction and significant bronchodilation effect. Controls were recruited at regular intervals from the same source population. The response rates of both cases (86%) and controls (80%) were good, and thus, any major selection bias is not likely. Smoking was assessed by questionnaire, but as the study was introduced to the participants as a study on environmental factors, with no specific focus on smoking, the likelihood of any major recall bias is small. Asthma was clinically defined, and all cases as well as controls with a previous diagnosis of asthma or long‐term use of asthma medications were excluded. In addition, for these analyses the ACOS cases were excluded. The definition of atopy was objective, based on presence of specific IgE‐antibodies in serum. For atopy we have no information on the age‐of‐onset and thus, we cannot draw conclusions on potential causal effects of smoking on atopy. The categories of occasional smokers and recent quitters were relatively small, and thus the interpretation of those results needs to be done with some caution.

#### 4.3.2 Synthesis with previous knowledge

Many, but not all,^29^, ^30^, ^31^, ^39^ previous studies have identified current and/or past smoking as risk factors for adult‐onset asthma, wheezing, or asthma attacks.^5^, ^6^, ^7^, ^8^, ^9^, ^10^, ^11^, ^12^, ^13^, ^14^, ^15^, ^16^, ^17^, ^18^, ^19^, ^20^, ^21^, ^22^, ^23^, ^24^, ^25^, ^26^, ^27^ Furthermore, atopic/allergic diseases and family history of atopy have also been reported to increase the risk of adult‐onset asthma.^6^, ^9^, ^10^, ^14^, ^17^, ^18^, ^21^, ^24^, ^27^, ^28^, ^29^, ^30^, ^31^, ^32^ However, only a few previous studies have evaluated if smoking has different effects on atopic and non‐atopic asthma in adults.^10^, ^14^, ^17^, ^32^ Two studies reported higher risk of adult‐onset asthma due to current smoking among non‐atopic than among atopic participants, OR 5.7 (95% CI 1.7–19.2) versus OR 1.8 (0.8–4.2)^10^ and OR 1.8 (1.3–2.6) versus OR 1.0 (0.6–1.9).^14^ In contrast, in two other studies current smoking increased the risk of asthma more among atopic than among non‐atopic participants, OR 1.4 (0.6–3.0) versus OR 0.7 (0.4–1.2)^17^ and OR 1.45 (0.81–2.61) versus OR 0.67 (0.4–1.15),^32^ but all effects were NS.

Considering the effect of ex‐smoking, two studies found a more pronounced effect among non‐atopic than atopic participants, OR 2.4 (1.5–3.9) versus OR not reported^14^ and OR 1.59 (1.00–2.54) versus OR 0.78 (0.36–1.65).^32^ In addition, one study reported increased, but NS, risk among both non‐atopic with OR 1.5 (0.9‐2.5) and atopic with OR 1.9 (0.8–4.2) participants.^17^ In summary, there was no consistency among the previous studies concerning the effect of smoking on atopic and non‐atopic adult‐onset asthma. Comparison of the results is difficult due to different definitions of exposures and outcomes being applied. Different smoking habits between the countries, for example, more common use of snus and less common cigarette smoking among Swedish versus Finnish men,^40^ could play some role for the observed differences. To our knowledge, our study is the first one that examines the effects of occasional and regular smoking and the effect of quitting time on subtypes of adult‐onset asthma, and studies genders separately. These specific details were found to be of importance, since if we had not considered them, we would have only found an increased risk of non‐atopic asthma in former smokers.

## 5 CONCLUSIONS

This study showed that tobacco smoking causes atopic and non‐atopic adult‐onset asthma with different dynamics. We show for the first time that gender modifies these effects. In women, relatively small amounts of regular smoking increase the risk of atopic asthma, and quitting reduces the asthma risk relatively fast. The effect of regular smoking on non‐atopic asthma risk is weaker, but this effect continues for longer after quitting, especially in men. This raises new questions about the underlying mechanisms of adult‐onset asthma and supports personalised approach in preventing smoking related damages.

## CONFLICT OF INTEREST

The authors declare no conflict of interests.

## AUTHOR CONTRIBUTIONS

Maritta S. Jaakkola and Jouni J.K. Jaakkola developed the study design; Taina K. Lajunen, Jouni J.K. Jaakkola and Maritta S. Jaakkola contributed to data analysis and interpretation of results; Taina K. Lajunen drafted the manuscript and Maritta S. Jaakkola and Jouni J.K. Jaakkola contributed substantially to revision of the manuscript. All authors read and approved the final manuscript.

## Supporting information

TABLE S1
