# Supplementary material for: Different effects of smoking on atopic and non‐atopic adult‐onset asthma
Source: Clin Transl Allergy. 2021 Oct 11;11(8):e12072. doi: 10.1002/clt2.12072 (PMC8504202; doi:10.1002/clt2.12072)
Supplement: Supplementary file 1 — TABLE S1 [file CLT2-11-e12072-s001.docx]

**Supplementary Table 1.** Distribution of the characteristics in the FEAS study among the cases of adult-onset asthma with and without a Phadiatop measurement.

| **Characteristic** | **Cases with Phadiatop results available**  **(atopic and non-atopic asthma cases)**  **n (%)** | **Cases without**  **Phadiatop results**  **n (%)** | **χ^2^-test p-value** |
| --- | --- | --- | --- |
| Total n | 463 | 32 |  |
| Gender |  |  | 0.263 |
| Men | 144 (31.1) | 13 (40.6) |  |
| Women | 319 (68.9) | 19 (59.4) |  |
| Age, years |  |  | 0.435 |
| 21-29 | 100 (21.6) | 8 (25.0) |  |
| 30-39 | 97 (21.0) | 8 (25.0) |  |
| 40-49 | 114 (24.6) | 4 (12.5) |  |
| 50-59 | 119 (25.7) | 11 (34.4) |  |
| 60-63 | 33 (7.1) | 1 (3.1) |  |
| Education ^†^ |  |  | 0.606 |
| No vocational schooling | 88 (19.1) | 9 (28.1) |  |
| Vocational course | 76 (16.5) | 5 (15.6) |  |
| Vocational institution | 138 (29.9) | 10 (31.3) |  |
| College-level education | 105 (22.8) | 4 (12.5) |  |
| University or corresponding | 54 (11.7) | 4 (12.5) |  |
| Pets at home sometimes |  |  | 0.901 |
| No | 135 (29.2) | 9 (28.1) |  |
| Yes | 328 (70.8) | 23 (71.9) |  |
| Any work exposure ^‡^ |  |  | 0.357 |
| No | 179 (38.7) | 15 (46.9) |  |
| Yes | 284 (61.3) | 17 (53.1) |  |
| Indoor mould exposure at work or at home |  |  |  |
| No visible mould or mould odour | 357 (77.1) | 20 (62.5) | 0.061 |
| Visible mould or mould odour | 106 (22.9) | 12 (37.5) |  |
| No visible mould | 409 (88.5) | 26 (81.25) | 0.253 (Fisher’s exact test) |
| Visible mould | 53 (11.5) | 6 (18.75) |  |
| No mould odour | 380 (82.1) | 23 (71.9) | 0.152 |
| Mould odour | 83 (17.9) | 9 (28.1) |  |
| Smoking ^¶^ |  |  |  |
| Never | 222 (48.26) | 14 (43.75) | 0.805 |
| Previous | 118 (25.65) | 8 (25.0) |  |
| Current | 120 (26.09) | 10 (31.25) |  |
| Never | 222 (48.26) | 14 (43.75) | 0.862 |
| Quit > 1 year ago | 90 (19.57) | 7 (21.88) |  |
| Quit < 12 months ago | 28 (6.09) | 1 (3.13) |  |
| Occasional | 27 (5.87) | 3 (9.38) |  |
| Regular | 93 (20.22) | 7 (21.88) |  |

The 26 ACOS cases were excluded from the present analyses.

^†^ Education missing for 2 asthma cases with Phadiatop results.

^‡^ Self-reported exposure to sensitisers, dusts and/or fumes.

^¶^ Smoking missing for 3 asthma cases with Phadiatop results.
